# Imaging screw dislocations at atomic resolution by aberration-corrected electron optical sectioning

**DOI:** 10.1038/ncomms8266

**Published:** 2015-06-04

**Authors:** H. Yang, J. G. Lozano, T. J. Pennycook, L. Jones, P. B. Hirsch, P. D. Nellist

**Affiliations:** 1 Department of Materials, University of Oxford, 16 Parks Road, Oxford OX1 3PH, UK; 2 EPSRC SuperSTEM Facility, STFC Daresbury, WA4 4AD, UK

## Abstract

Screw dislocations play an important role in materials' mechanical, electrical and optical properties. However, imaging the atomic displacements in screw dislocations remains challenging. Although advanced electron microscopy techniques have allowed atomic-scale characterization of edge dislocations from the conventional end-on view, for screw dislocations, the atoms are predominantly displaced parallel to the dislocation line, and therefore the screw displacements are parallel to the electron beam and become invisible when viewed end-on. Here we show that screw displacements can be imaged directly with the dislocation lying in a plane transverse to the electron beam by optical sectioning using annular dark field imaging in a scanning transmission electron microscope. Applying this technique to a mixed [***a***+***c***] dislocation in GaN allows direct imaging of a screw dissociation with a 1.65-nm dissociation distance, thereby demonstrating a new method for characterizing dislocation core structures.

## Abstract (web summary)

Although screw dislocations impact on the properties of various engineering materials, their investigation on the atomic scale has been challenging. Here, the authors use optical sectioning in a scanning transmission electron microscope to achieve direct imaging of screw displacements around a screw dislocation core in GaN.

## Graphical abstract

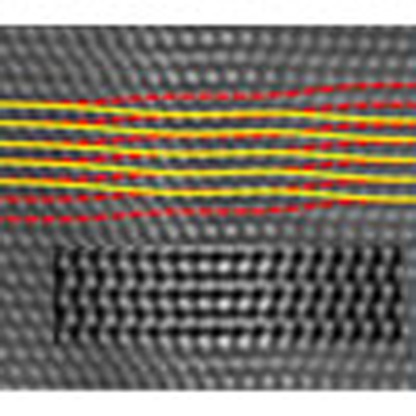

Edge dislocations are characterized by columns of atoms in tension or in compression in a plane normal to the dislocation, above and below the slip plane. Such dislocations are conventionally viewed end-on at high resolution in transmission electron microscopes because the tensile and compressive displacements are clearly visible^1^,^2^,^3^,^4^,^5^. Screw dislocations are characterized by equivalent atoms in columns parallel to the screw dislocations being displaced along the screw axis to form a helicoid, which cannot be observed end-on because the displacements are parallel to the viewing direction. Such screw dislocations do however show small rotational displacements in thin foils due to surface stress relaxations known as the Eshelby twist^6^, and these can affect both the contrast and peak positions in high-resolution images^7^,^8^,^9^,^10^. Here we show that the helicoidal displacements around a screw can be imaged directly with the dislocation lying in a plane transverse to the electron beam by optical sectioning using annular dark field (ADF) imaging in a scanning transmission electron microscope (STEM). This novel technique is illustrated by application to the screw component [***c***] in the dissociation of a mixed [***a***+***c***] dislocation in GaN, which has only been observed end-on previously^11^.

Because of the relatively large lattice mismatch with the substrate, GaN films grown parallel to (0001) generally contain threading dislocations, and during growth the dislocations tend to align themselves close to the [0001] direction. Three types of dislocations are found: edge, screw and mixed dislocations with Burgers vectors [***a***], [***c***] and [***a+c***], respectively. The [***a+c***] mixed dislocations are frequently found to be dissociated^4^,^5^, and a recent study in ref. 11 showed that the edge component dissociates by climb according to the reaction [***a***]=½[***a***]+½[***a***] to form a fault lying in the 
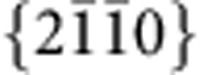
 plane (***a***-plane). Since the ½[***a***] shift would give rise to Ga–Ga and N–N bonding and therefore a very high-energy interface, an additional ½[***c***] shift along [0001] was proposed so that correct Ga–N pairings across the interface are possible. This implies that the screw component is also dissociated according to [***c***]=½[***c***]+½[***c***], and therefore the total dissociation reaction proposed is [***a***+***c***]=½[***a***+***c***]+½[***a***+***c***], and the two partials are bounding a fault with a structure similar to the one proposed by Drum in ref. 12, with the dissociation distance of ∼1.8 nm measured on the electron micrographs^5^,^11^.

The development of aberration correctors in (S)TEM (transmission electron microscopy) has led to a dramatic improvement in spatial resolution in both STEM^13^,^14^ and conventional TEM^15^; however, there is also a considerable reduction in the microscope depth of field, which in a state of the art microscope may be just a few nanometres. This creates an opportunity to focus at specific depths within the sample and to extract information from the layers at those depths—a process referred to as ‘optical sectioning'. Achieving the full-depth resolution available for a general sample requires a confocal configuration; however, at atomic resolution a conventional STEM configuration can be used^16^,^17^.

## Results

### Image simulations of the screw displacements

**Figure 1:**
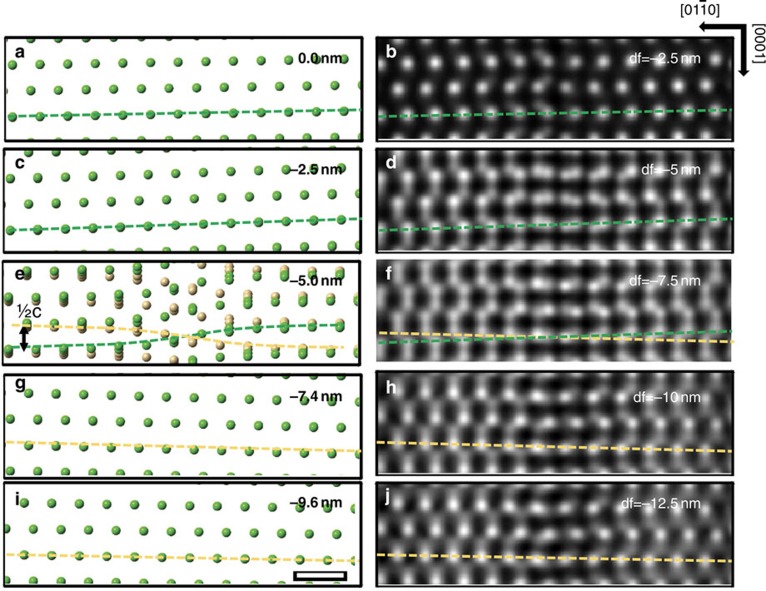
Depth-dependent screw displacements in a GaN c-type screw dislocation. The positions of the Ga atoms in single layers of 
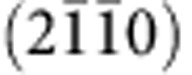
 planes at various depths in a structure model of a GaN c-type screw dislocation (left) and simulated ADF focal series images (right). The screw dislocation is along [0001] and located at a depth of −5 nm, and the single 
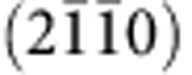
 layers are located at **a** 0, **c**−2.5, **e**−5.0, **g**−7.4 and **i**−9.6 nm below the top surface, respectively. In this work depths into the crystal are given by negative values and are measured relative to the entrance surface. Similarly, negative defocus denotes under focusing so that the focal plane moves below the entrance surface into the crystal. Dashed lines show the shearing of planes due to the screw. In **e**, the two planes immediately above and below the dislocation line are overlaid and indicated by different colours. Owing to a channelling pre-focusing effect, each simulated focal series image on the right (**b**,**d**,**f**,**h**,**j**) has a focus set ∼2 nm lower in depth than the corresponding layer of model on the left. The scale bar, 0.5 nm.

Figure 1 shows that the shearing of atomic planes around a screw dislocation can be imaged in a transverse view of the dislocation line using optical sectioning. A model of the ***c***-type screw dislocation along [0001] in GaN is used, and atomic positions in single layers parallel to 
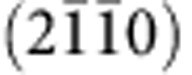
 planes normal to the electron beam are shown at various depths relative to the dislocation line. The atomic positions are calculated for an infinite crystal assuming isotropic elasticity (see Supplementary Note 1), an assumption based on the fact that GaN is isotropic on the basal plane^18^. In the TEM thin foil, the surface relaxation due to image dislocations was considered, and the displacements due to such image dislocations were found to be negligible compared with the screw shear of up to ½[***c***] (see Supplementary Fig. 1 and Supplementary Note 2). Far from the dislocation core, the screw displacements vary slowly across the field of view as expected from the lower shear strain that exists further from the dislocation core (Fig. 1a,c,g,i). Close to the core (Fig. 1e), screw displacements with a rapidly varying shear of the (0002) planes are observed giving an apparent displacement of ½[***c***] across the dislocation core (as expected for a total Burgers vector of [***c***]).

**Figure 2:**
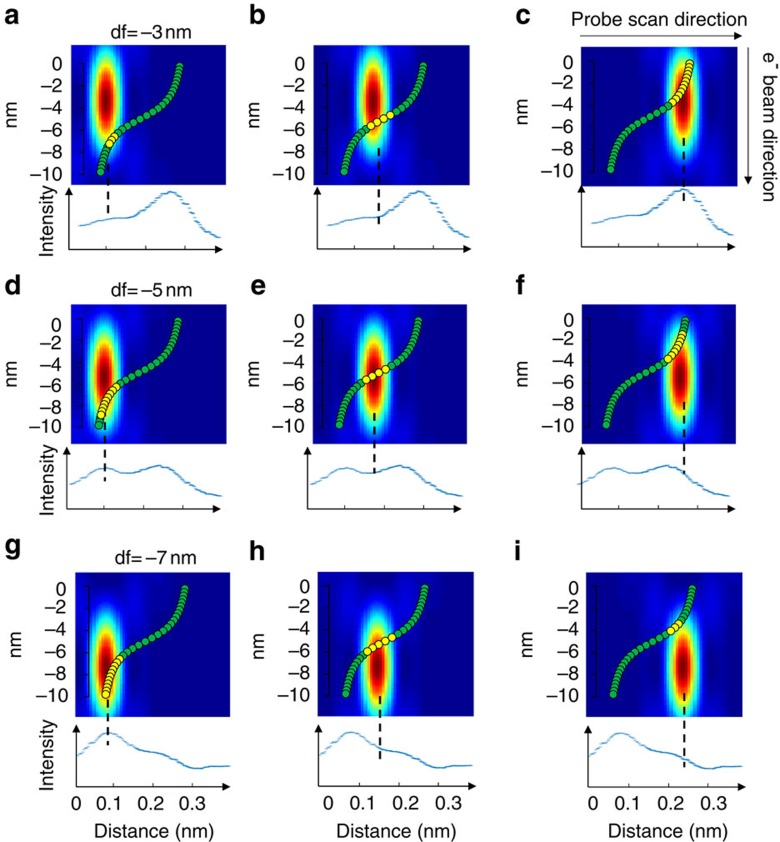
Schematic illustration of the image contrast in focal series ADF images of a screw dislocation. The electron probe is focused at different depths and positions relative to a column, which has an arctangent shape due to the screw displacements. When the probe is focused at (**a**–**c**) −3 nm, (**d**–**f**) −5 nm and (**g**–**i**) −7 nm below the specimen entrance surface, different sections of the column are located close to the brightest part of the probe (atoms indicated by yellow colour). The peak image intensity occurs when the largest number of atoms lie in the most intense region of the elongated probe, as shown from the intensity line profiles of simulated focal series ADF images. This suggests that, despite the effects of dynamical scattering and channelling, the image contrast in focal series images are intuitively interpretable for identifying column locations and measuring screw displacements.

In the simulated ADF images (100 keV beam energy, probe convergence semi-angle 30 mrad), the peaks associated with the atomic columns become double peaks as the focal plane approaches the dislocation depth, and the relative intensities of these peaks change with defocus (Fig. 1d,f,h). (For a smaller convergence semi-angle, the depth-dependent information cannot be observed, see Supplementary Fig. 2 and Supplementary Note 3.) The evolution of double peak intensities with changing defocus can be explained in terms of the elongated nature of the illuminating probe along the electron propagation direction. When the screw dislocation lies transverse to the electron beam, the atomic columns follow an arctangent shape (Fig. 2). As the probe is scanned at various depths, the strength of electron scattering will depend on the number of atoms included in the most intense part of the illuminating probe. Because of the elongated probe, the section of an atomic column that is aligned with the beam will have a stronger projected intensity than the more sheared sections. Figure 2 explains why a column appears as a double peak with comparable intensities when focused close to the core, and why the relative intensity of double peaks is reversed when the focus is changed from above to below the core. One of the peaks of the doublet arises from the column just above the screw dislocation, the other from the column just below. The shear across the screw, and therefore the magnitude of the Burgers vector, can be determined from the positions of, say, the line of strong peaks across the screw (or equally from the weak peaks). The shear measured using the stronger peaks appears reversed when the focal plane changes from above to below the core allowing us to determine the sign of the screw Burgers vector. An approximately 2-nm pre-focusing effect due to electron channelling is taken into account^19^ in order to determine the correct depths of focal series images.

### STEM optical sectioning of GaN dislocations at the atomic scale

**Figure 3:**
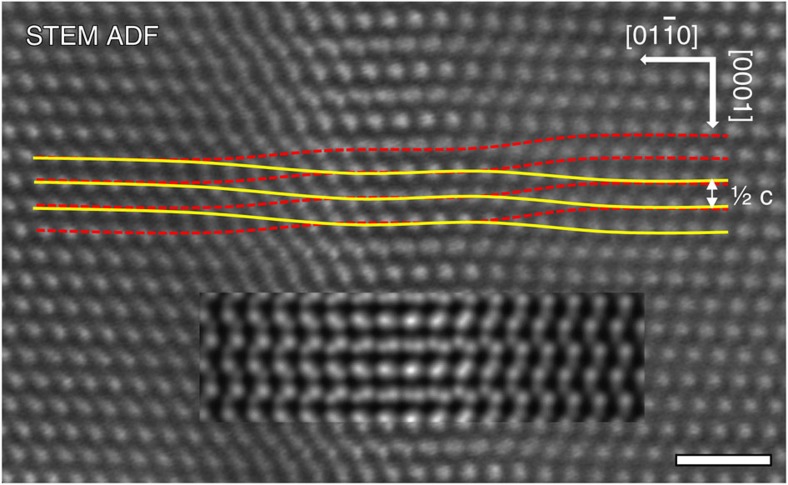
Experimental and simulated ADF image of a dissociated dislocation lying perpendicular to the electron beam. The experiment STEM ADF image is the average of 64 images (see Supplementary Note 4). The screw displacements associated with each of the partial dislocations can be observed, as indicated by the overlaid solid and dashed lines following the closer-to-focus stronger-intensity peaks and further-from-focus weaker-intensity peaks, respectively. A simulated image (inset) of the isotropic elastic model of a ½[***a***+***c***]+½[***a***+***c***] dissociated dislocation with a 1.65-nm dissociation distance is overlaid. The simulation was performed with the beam focused at 5 nm below the top entrance surface of a 10-nm-thick foil. The scale bar, 1 nm.

The optical sectioning technique is now applied to understand the mixed dislocations in GaN. An [***a***+***c***] dislocation lying approximately along [0001] in the plane of the sample parallel to 
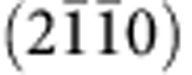
 was identified by the weak-beam technique; however, no dissociation was apparent in this experiment. A focal series of high-resolution STEM ADF images was acquired. The 30-mrad probe convergence aperture gives a depth of field of 7.28 nm. Focused close to the dislocation core (Fig. 3), the (0002) planes both above and below the dislocation core, sheared in opposite directions, are simultaneously resolved, with the higher-intensity planes (indicated by solid curves) corresponding to atoms located closer to focus and the low-intensity planes (dashed curves) being further away from focus and on the opposite side of the core. The rapid shear occurs along two distinct bands suggesting that the screw dislocation is dissociated into two partials. The magnitude of the shear across each partial is ¼[***c***], and therefore the Burgers vector of each screw partial is ½[***c***], giving a total screw Burgers vector of [***c***] as expected for GaN. The screw shear is further confirmed by quantifying peak positions from the ADF focal series (Supplementary Fig. 3 and Supplementary Note 4). The screw component [***c***] of the [***a***+***c***] dislocation therefore dissociates according to the reaction [***c***]=½[***c***]+½[***c***] as predicted on structural and elastic energy grounds^11^. Combining this with the dissociation of the edge component [***a***] observed in end-on images, the overall dissociation reaction is therefore experimentally determined to be [***a***+***c***]=½[***a***+***c***]+½[***a***+***c***].

### Image simulations of dissociated dislocation models

**Figure 4:**
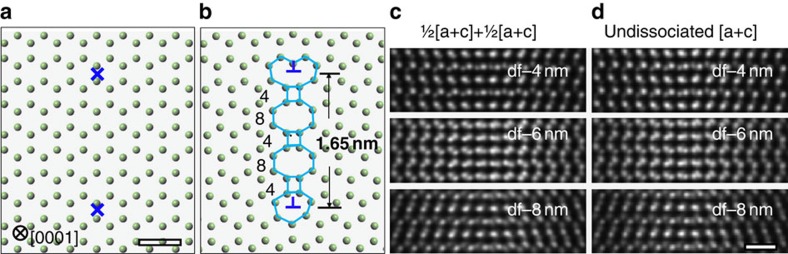
The structure model and simulated focal series images of the dissociated dislocation. (**a**) The dots represent the positions of the mixed Ga and N columns viewed along [0001]. The core positions of the two partials are located as indicated, and result in an atomic structure, shown in **b**, that matches the image shown in Fig. 2 of ref. 11. The resulting fault has a 4/8/4/8/4-ring structure, similar to the Drum fault, and a 1.65-nm dissociation distance. Simulated focal series ADF images of the dissociated ½[***a***+***c***]+½[***a***+***c***] and the undissociated [***a***+***c***] model viewed along 
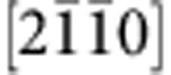
 are shown in **c**,**d**, respectively. The scale bar in the simulated image, 0.5 nm.

To measure the dissociation distance accurately, structure models of the dissociated dislocation were built and multislice frozen phonon image simulations were carried out. The atomic positions in the dissociated ½[***a*****+*****c***]+½[***a*****+*****c***] dislocation models were determined using an isotropic elastic strain field in an infinite crystal, assuming that the cores of the screw and edge partials are at the same positions. The positions of the cores of the edge dislocation partials were determined by considering several candidate positions^20^ (Supplementary Fig. 4). By placing the cores at the midpoint between neighbouring columns (Fig. 4a), the resultant dissociated dislocation structures (Fig. 4b) viewed along [0001] match reasonably well the core structures shown in Fig. 2 of ref. 11, although this latter image was complicated because of the presence of kinks and jogs. The core structures of the partials in Fig. 4b also agree with both the experiments and atomistic simulations in Fig. 2(i,iii) of ref. 5. The Drum fault between the partials observed experimentally in Fig. 2 of ref. 11 consists of a 4/8/4/8/4-ring structure as in Fig. 4b of this paper, but modified by kinks^11^. In the ADF images in Fig. 3 of ref. 11 and Fig. 2(i) of ref. 5, the partial dislocations are closer together bounding a 4/8/4-ring fault structure. All four possible dissociation distances below 3 nm, consisting of different numbers of 4/8 rings in the Drum fault, were considered (Supplementary Fig. 5). The [***a***+***c***] undissociated screw model was also built for comparison. Simulated focal series images of both the dissociated screw model with a 1.65-nm dissociation distance and the undissociated screw model are shown in Fig. 4c,d.

### Quantification of the screw dissociation distance

**Figure 5:**
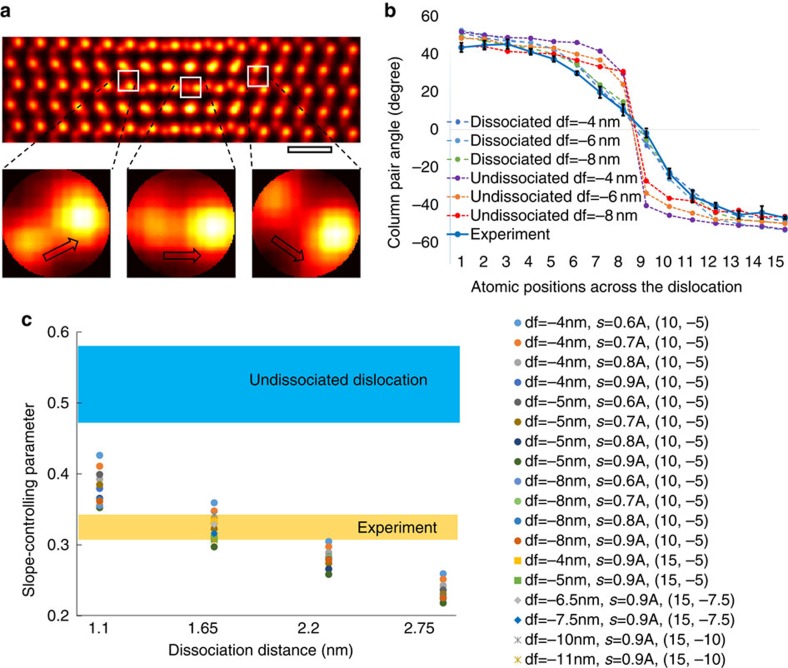
Determining screw dissociation through quantitative analysis of screw displacements near the dislocation. (**a**) The images in the fault region consist of pairs of closely spaced peaks whose relative positions correlate with the amount of screw displacement. A Radon transform is used to measure changes in the column pair angles across the dislocation (see Supplementary Note 5). (**b**) The column pair angles have been measured for both the experimental and simulated images of dissociated screw (1.65-nm dissociation distance) and undissociated screw dislocations, and a good match is found between experiment and the dissociated screw column pair angles, confirming the experimental observation of a dissociated screw. The error bar in **b** corresponds to the s.d. of multiple measurements from the entire experiment image shown in Fig. 3. (**c**) The rate of angle change across the dislocation depends on the dissociation distance; therefore, by fitting sigmoid functions to the angle plots, and comparing the fitted slope-controlling parameter in the sigmoid functions of the experimental image with those of simulated images of different possible dissociation distances, the dissociation distance of 1.65 nm clearly gives the best agreement. The robustness of this quantification method against probe defocus and source size is examined across all four dissociation distances, and in the case of 1.65-nm dissociation distance, results of different specimen thicknesses and dislocation depths inside the crystals are also included in the plot in **c**. The legend in **c** shows the value of the parameters, for example, ‘df=−4 nm, s=0.6A, (10, −5)' means defocus −4 nm, source size 0.6 Angstrom, (10, −5) means the specimen thickness being 10 nm and the depth of dislocation below the top entrance surface being −5 nm. The scale bar in **a**, 0.5 nm.

The key difference between the images of the dissociated screw (Fig. 4c) and undissociated screw (Fig. 4d) is the rate at which the screw displacements change across the dislocations. The screw displacements in the fault region between two dissociated screw partials change more slowly than corresponding displacements in an undissociated screw. Instead of measuring the screw displacements directly, the structure of the GaN crystal allows us to adopt the following more sensitive method, which is also robust to scan distortions. As explained further in the Supplementary Note 5, the images in the fault region consist of pairs of closely spaced peaks (see Fig. 5a) whose relative positions correlate with the amount of screw displacement. The screw displacement can be quantitatively analysed by measuring the change in the orientation of the line joining the column pair across the dislocation (Fig. 5a). Details of the angle measurement using a Radon transform are included in the Supplementary Fig. 6 and Supplementary Note 5. This approach provides a method for measuring the screw displacements that does not rely on correlating displacements over large distances in the image, which would be much more sensitive to scan distortions than the very local information used here.

Measurements on simulated images clearly show a more rapid change in the angles in the undissociated dislocation than in the dissociated dislocation, with the most significant difference found in the fault region between the two dissociated screw partials (Fig. 5b). Moreover, the rate of change in screw displacements across the dislocations depends on the dissociation distance, with the rate increasing with decreasing dissociation distance, and to a lesser extent on the probe defocus. By fitting the angle plots with four-parameter sigmoid functions, the rate can be represented by the slope-controlling parameter of the sigmoid functions (see Supplementary Note 5). As shown in Fig. 5c, by comparing the fitted slope-controlling parameter from both experiment and simulated images of the undissociated and dissociated dislocations with different dissociation distances, the experimental measurements (taking into account a s.d. coming from measurements of several lattice planes) agree with a 1.65-nm dissociation distance under various focusing conditions. It is clear from Fig. 5b that for this dissociation distance the angles measured from the experimental image match those from the simulated images over the whole range plotted. Figure 5c also shows that the significant difference in the fitted slope-controlling parameters between the undissociated and dissociated dislocations suggests that the sensitivity of the method should be sufficient to detect a screw dissociation with a dissociation distance as small as 1.1 nm in GaN. The sensitivity demonstrated here is sufficient to distinguish changes in dissociation distance of 0.55 nm. The structure shown in Fig. 2 in the paper described in ref. 11 corresponds to a dissociation distance of 1.65 nm as shown in Fig. 4 of this paper. The value of 1.8 nm reported in ref. 11, obtained by direct measurement on the image, is inaccurate because of the uncertainty in determining the termination of half planes of two edge partials from the STEM micrographs.

## Discussion

The results presented here confirm the predicted dissociation of the [***a***+***c***] mixed dislocation in GaN along ***c*** into ½[***a***+***c***]+½[***a***+***c***] bounding a Drum fault^11^. It should be noted that specimens used here and in the previous work^11^ involved different synthesis methods but show the same dissociation reaction for the GaN [***a***+***c***] mixed dislocations. These results also show that the characteristic helicoid structure of screw dislocations can be imaged more generally in cross-section using the STEM optical sectioning technique, which provides a direct measurement of the screw Burgers vectors. This might be compared with previous efforts using either convergence beam electron diffraction patterns^21^, conventional STEM imaging, which has only been demonstrated in case of an enormously large Burgers vector^22^, or the use of forbidden reflections^23^,^24^. Moreover, the measured dissociation distance of 1.65 nm is at the borderline of what might be observable by the conventional weak-beam technique. Weak-beam requires an objective aperture about an order of magnitude smaller than the one used here, and therefore the diffraction-limited spatial resolution of weak-beam imaging is close to the dissociation distance measured here. Furthermore, both we and previous authors^4^ have attempted weak-beam imaging on this type of dislocation and have failed to detect this dissociation. For these reasons, and because our analysis suggests that a dissociation distance as small as 1.1 nm could be detected for GaN (see Fig. 5), we believe that this approach offers a high-resolution alternative to weak-beam imaging for the screw components of a dislocation.

The method used here to measure the dissociation distance quantitatively is specific to the structure of GaN, and other structures may require other approaches to measure atomic displacements in STEM images, including, for example, careful correction of scan distortion. There will be a dislocation depth beyond which clear images cannot be formed because of probe scattering by the crystal above the dislocation, but Fig. 5 shows that our method is robust at least to a dislocation depth of 10 nm in a foil of 15-nm thickness (see Supplementary Fig. 7 and Supplementary Note 5). Investigations of the limits of this approach require further work; however, we note that improvements in electron optics will continue to offer higher numerical apertures and smaller depths of field leading to expected improvements in the sensitivity of the optical sectioning approach.

## Methods

### Specimen preparation

The sample studied consists of a thin GaN foil parallel to (0001). A 1-μm-thick GaN film was grown at 1,000 °C on a sapphire substrate by metalorganic vapour phase epitaxy. The TEM specimen was prepared by mechanical grinding and polishing, followed by ion milling using a Gatan Precision Ion Polishing System.

### Scanning transmission electron microscopy

The high-angle annular dark field STEM images were obtained using the Nion UltraSTEM100 electron microscope at SuperSTEM, Daresbury, UK. This microscope is equipped with a field emission gun operated at 100 kV, and a Nion fifth-order spherical aberration (Cs) corrector. A probe convergence semi-angle of 30 mrad was used to achieve a 7.28-nm depth of field (see Supplementary Fig. 8 and Supplementary Note 3). The images were captured by an ADF detector with a collection angle ranging from 90 to 200 mrad.

### Multislice frozen phonon image simulations

To simulate the STEM ADF images, we have used the multislice frozen phonon approach implemented in the QSTEM software using the same imaging parameters (accelerating voltage and convergence angle) as those used in the experimental work^25^. Thermal diffuse scattering is introduced using the frozen phonon method and up to 30 configurations of thermal displacements of atoms are averaged in the simulated images.

## Additional information

**How to cite this article:** Yang, H. *et al*. Imaging screw dislocations at atomic resolution by aberration-corrected electron optical sectioning. *Nat. Commun.* 6:7266 doi: 10.1038/ncomms8266 (2015).

## Supplementary Material

Supplementary Information Supplementary Figures 1-8, Supplementary Notes 1-5 and Supplementary References

## References

[b1] Menter J. W. The direct study by electron microscopy of crystal lattices and their imperfections. Proc. R. Soc. A 236, 119–135 (1956).

[b2] Hirsch P., Cockayne D., Spence J. & Whelan M. 50 years of TEM of dislocations: past, present and future. Philos. Mag. 86, 4519–4528 (2006).

[b3] Xin Y. . Direct observation of the core structures of threading dislocations in GaN. Appl. Phys. Lett. 72, 2680–2682 (1998).

[b4] Arslan I., Bleloch A., Stach E. A. & Browning N. D. Atomic and electronic structure of mixed and partial dislocations in GaN. Phys. Rev. Lett. 94, 025504 (2005). 15698191 10.1103/PhysRevLett.94.025504

[b5] Rhode S. K. . Mg doping affects dislocation core structures in GaN. Phys. Rev. Lett. 111, 25502 (2013). 10.1103/PhysRevLett.111.025502 23889417

[b6] Eshelby J. D. & Stroh A. N. Dislocation in thin plates. Philos. Mag. 42, 1401–1405 (1951).

[b7] Mendis B. G., Mishin Y., Hartley C. S. & Hemker K. J. Use of the Nye tensor in analyzing HREM images of bcc screw dislocations. Philos. Mag. 86, 4607–4640 (2006).

[b8] Lozano J. G. . Direct observation of depth-dependent atomic displacements associated with dislocations in gallium nitride. Phys. Rev. Lett. 113, 135503 (2014). 25302902 10.1103/PhysRevLett.113.135503

[b9] Gröger R. . Effect of Eshelby twist on core structure of screw dislocations in molybdenum: atomic structure and electron microscope image simulations. Philos. Mag. 91, 2364–2381 (2011).

[b10] Sigle W. High-resolution electron microscopy and molecular dynamics study of the (a/2)[111] screw dislocation in molybdenum. Philos. Mag. A 79, 1009–1020 (1999).

[b11] Hirsch P. B. . The dissociation of the [ a+c ] dislocation in GaN. Philos. Mag. 93, 3925–3938 (2013).

[b12] Drum C. M. Intersecting faults on basal and prismatic planes in aluminium nitride. Philos. Mag. 11, 313–334 (1965).

[b13] Batson P. E., Dellby N. & Krivanek O. L. Sub-ångstrom resolution using aberration corrected electron optics. Nature 418, 617–620 (2002). 12167855 10.1038/nature00972

[b14] Nellist P. D. . Direct sub-angstrom imaging of a crystal lattice. Science 305, 1741 (2004). 15375260 10.1126/science.1100965

[b15] Haider M. . A spherical-aberration-corrected 200 kV transmission electron microscope. Ultramicroscopy 75, 53–60 (1998).

[b16] Nellist P. D. & Wang P. Optical sectioning and confocal imaging and analysis in the transmission electron microscope. Annu. Rev. Mater. Res. 42, 125–143 (2012).

[b17] Van Benthem K. . Three-dimensional imaging of individual hafnium atoms inside a semiconductor device. Appl. Phys. Lett. 87, 034104 (2005).

[b18] Hirth J. P. & Lothe J. Theory of Dislocations Wiley (1982).

[b19] Cosgriff E. C. & Nellist P. D. A Bloch wave analysis of optical sectioning in aberration-corrected STEM. Ultramicroscopy 107, 626–634 (2007). 17306928 10.1016/j.ultramic.2006.12.004

[b20] Béré A. & Serra A. Atomic structure of dislocation cores in GaN. Phys. Rev. B 65, 205323 (2002).

[b21] Tizei L. H. G. . Enhanced Eshelby twist on thin wurtzite InP nanowires and measurement of local crystal rotation. Phys. Rev. Lett. 107, 195503 (2011). 22181625 10.1103/PhysRevLett.107.195503

[b22] Li F. . Direct observation of a screw dislocation normal to the beam by Z-contrast STEM. J. Am. Ceram. Soc. 95, 466–468 (2012).

[b23] Spence J. C. H. . Imaging dislocation cores—the way forward. Philos. Mag. 86, 4781–4796 (2006).

[b24] Kolar H. R., Spence J. C. H. & Alexander H. Observation of moving dislocation kinks and unpinning. Phys. Rev. Lett. 77, 4031–4034 (1996). 10062370 10.1103/PhysRevLett.77.4031

[b25] Koch C. *Determination of Core Structure Periodicity and Point Defect Density Along Dislocations*. PhD thesis, Arizona State Univ. (2002).

