# Supplementary material for: Imaging screw dislocations at atomic resolution by aberration-corrected electron optical sectioning
Source: Nat Commun. 2015 Jun 4;6:7266. doi: 10.1038/ncomms8266 (PMC4468905; doi:10.1038/ncomms8266)
Supplement: Supplementary Information — Supplementary Figures 1-8, Supplementary Notes 1-5 and Supplementary References [file ncomms8266-s1.pdf]

Supplementary Figures

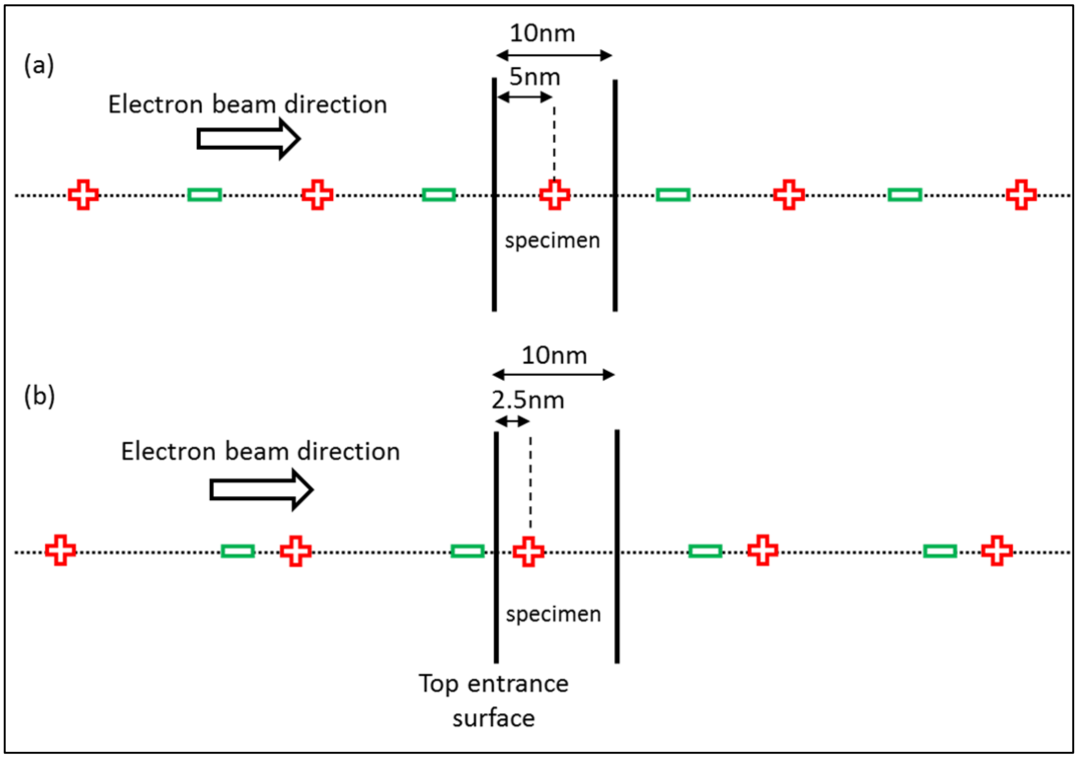

Supplementary Figure 1: **Schematic illustration of image dislocations considered in the TEM thin foil for building the structure models**. Two situations are considered: the screw dislocation lying **(a)** 5nm and **(b)** 2.5nm below the surface of a 10nm thick foil. A series of 4 image dislocations for each side of the foil are included in the screw displacement calculations. The "+" and "–" signs indicate screw dislocations with opposite Burgers vector.

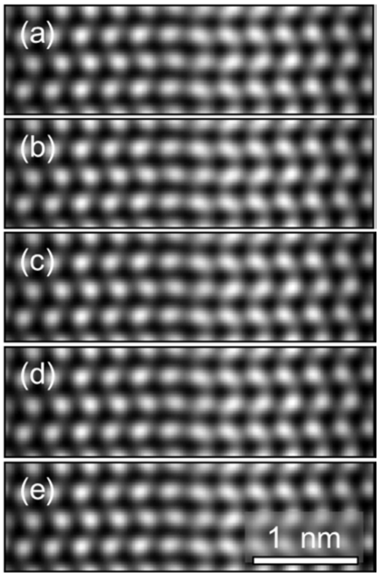

Supplementary Figure 2: Simulated focal series STEM ADF images of a GaN *c*-type screw dislocation. The images were simulated at various defocus values using an uncorrected microscope with a 15mrad convergence semi-angle. The screw dislocation is along [0001] and located at a depth of -5nm inside a 10nm thick sample. The defocus values from (**a**) to (**e**) are 0nm, -2nm, -4nm, -6nm and -8nm at depths below the top entrance surface, respectively.

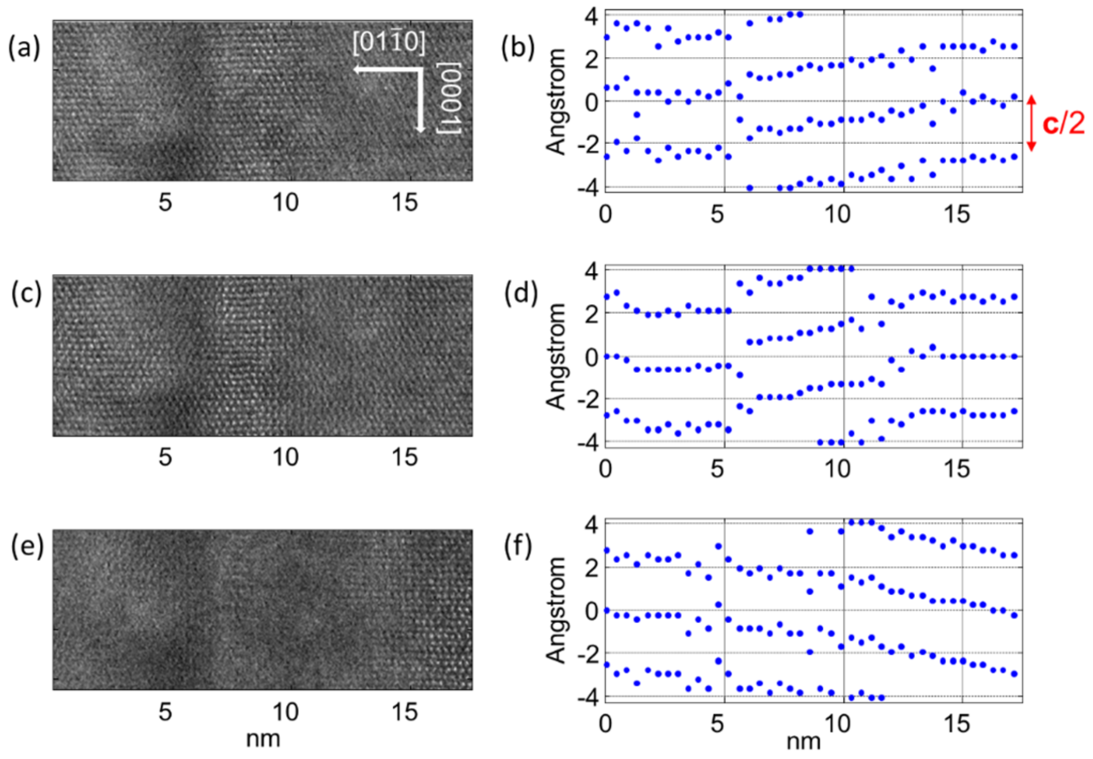

Supplementary Figure 3. Optical sectioning STEM ADF imaging of a dissociated [*a* + *c*] dislocation. The dislocation is viewed along [0001] viewed perpendicular to the dislocation line along [$2\bar{1}\bar{1}0$] with the focus (**a**) above, (**c**) close to, and (**e**) below the dislocation core. The shearing of the (0002) planes across the width of the image with respective to the bulk lattice are quantified for each defocus condition, and shown in (**b**), (**d**) and (**f**) side by side to (**a**) (**c**) and (**e**). Each point in the plots (**b**, **d**, **f**) represents a relative displacement between the intensity profile and the reference intensity profile, which gives a peak cross-correlation value. Due to the periodic nature of the intensity profile of the (0002) planes, there exist multiple peak values of cross-correlation when the amount of displacement is offset by multiples of ½ ***c***. In the plots, only the amount of displacement values that are less than the screw Burgers vector ***c*** are shown.

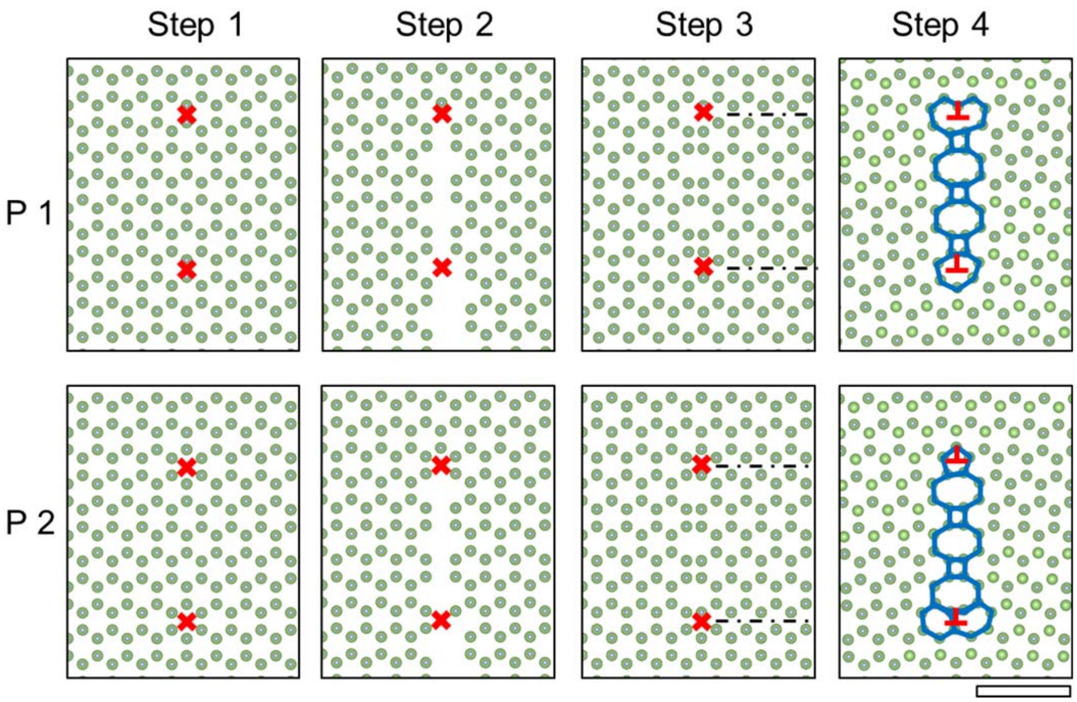

Supplementary Figure 4. Steps for generating the ½ [*a* + *c*] +½ [*a* + *c*] dissociated dislocation models. In step-1, the core positions of the dislocation partials located at either between a pair of atomic columns (P1) or at the centre of hexagonal rings (P2) are considered. To introduce the edge dislocation, one half ***a***-plane below the upper edge partial and another half ***a***-plane below the lower edge partial are deleted in step-2, and the atomic plane below each partial are shifted by ½ ***a*** in step-3. Assuming the screw partial positions coincide with the edge partials, isotropic elastic displacements of atomic positions due to edge and screw partial dislocations are introduced using Eqs. (S1-3) in step-4 to obtain the elastic models. The scale bar is 1nm.

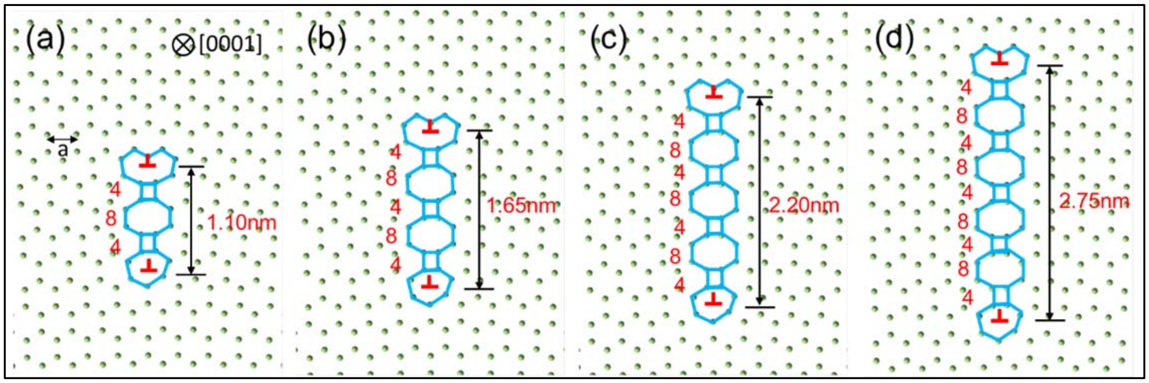

Supplementary Figure 5: **Dissociated ½ [*a* + *c*] +½ [*a* + *c*] dislocation models with four different dissociation distances**, (a) 1.10nm, (b) 1.65nm, (c) 2.20nm and (d) 2.75nm. The fault structure between two partials contain different numbers of 4/8 ring structures. In these four structure models, the core positions of the partials are all located midway between two neighbouring columns.

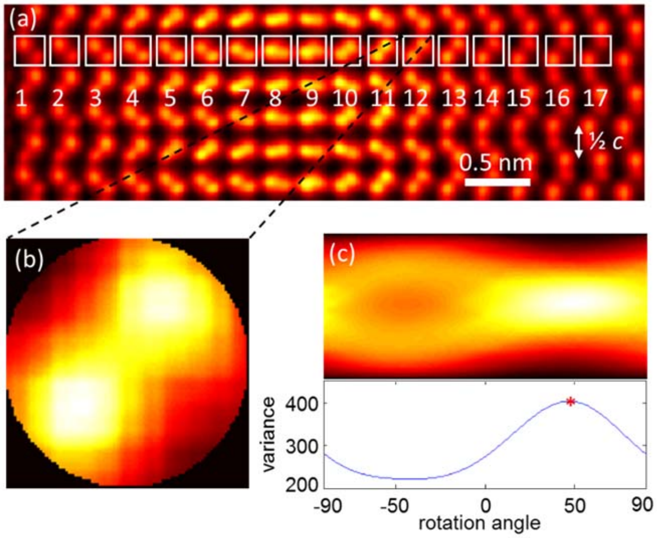

Supplementary Figure 6. **Radon transform angle measurement on the pairs of closely-spaced column peaks in ADF images**. Regions where the radon measurements were performed are indicated in white rectangular boxes in (a). One of these pairs of column peaks is magnified in (b), and for the Radon transform a circular mask is applied to the image crop in (b). The Radon transform of the image in (b) from -90° to 90° is shown in (c), where the intensity along the vertical direction represents the 1-dimensional projected intensity of image in (b) as a function of rotation angle. The variance of Radon projected intensity profile is calculated at each rotation angle and shown in (c). When the Radon angle is aligned with the column pair, the Radon transform shows a maximum variance, as can be seen near 50° in (c).

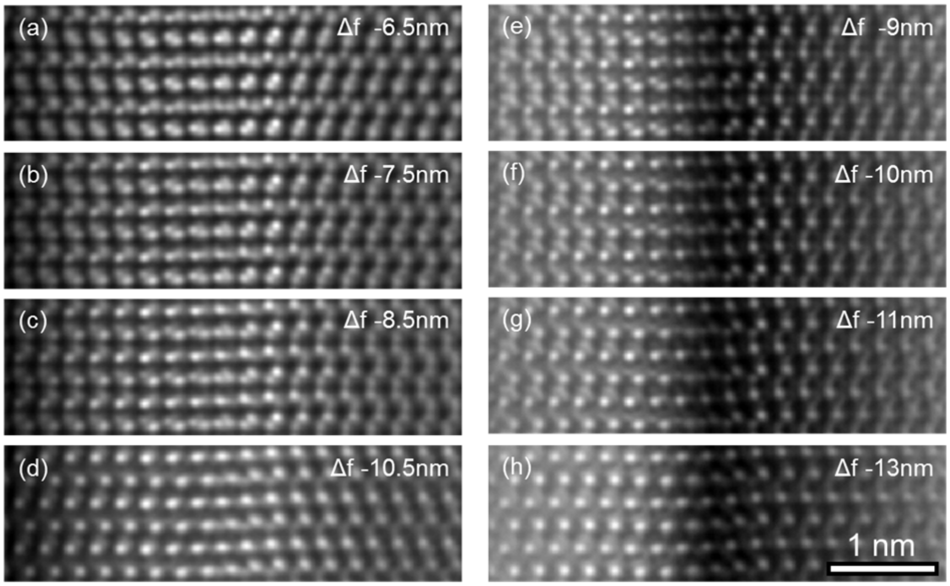

Supplementary Figure 7. **Simulated focal series ADF images of the ½ [*a* + *c*] +½ [*a* + *c*] dissociated dislocation model at two different thicknesses.** (a)-(d) has a total specimen thickness of 15nm and the dislocation is located -7.5nm below the top entrance surface; (e)-(h) has a total thickness of 20nm and the dislocation is located -10nm below the top entrance surface. In both cases the dissociation distance is 1.65nm.

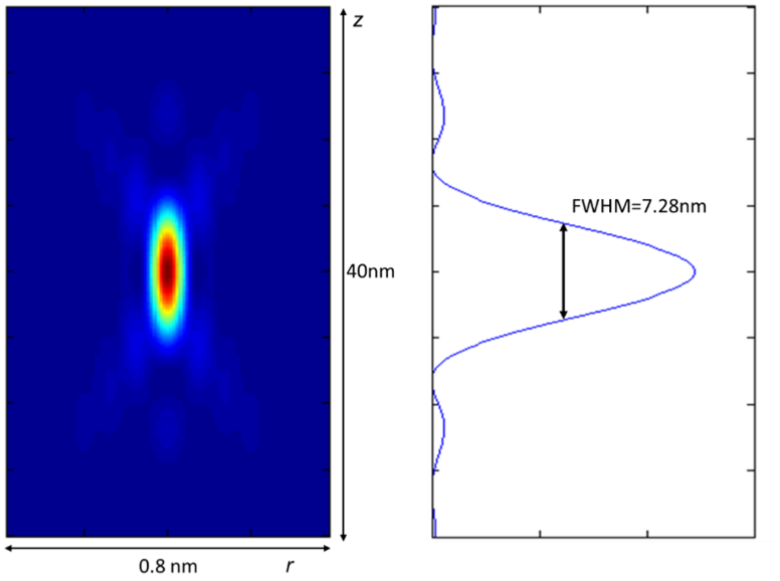

Supplementary Figure 8. Simulated STEM probe intensity profile along the beam direction. (Left) Simulated probe intensity plotted in the z-r plane for a 100-kV STEM with 30-mrad semi-angle of convergence. (Right) The intensity plotted along the optical axis (z-direction) at the centre of the STEM probe, showing a FWHM of 7.28nm as expected from the formula in Eq.S4.

Supplementary Note 1. Isotropic elastic structure models in an infinite crystal

Isotropic elastic equations described by Hirth and Lothe [1] are used to build GaN $\boldsymbol{c}$-type screw dislocation and dissociated $[\boldsymbol{a} + \boldsymbol{c}]$ dislocation models. The displacements of atoms due to an edge dislocation along [0001] are given by:

$$u_r = \frac{b_e}{2\pi}\left[-\frac{(1-2v)}{2(1-v)}\sin\theta_e \, lnr + \frac{\sin\theta_e}{4(1-v)} + \theta_e\cos\theta_e\right] \qquad \text{(S1)}$$

$$u_\theta = \frac{b_e}{2\pi}\left[-\frac{(1-2v)}{2(1-v)}\cos\theta_e \, lnr - \frac{\cos\theta_e}{4(1-v)} - \theta_e\sin\theta_e\right] \qquad \text{(S2)}$$

, where u_r and u_θ describe the displacements in polar coordinates, r and θ_e, and v is Poisson's ratio. For a typical c-axis-oriented GaN film, $v = 0.183$ is used.[2] The angle, θ_e, is within the range [0,2π] and the zero degree angle is defined along the Burgers vector. The Burgers vector for the edge component at each partial dislocation is $\boldsymbol{b_e} = ½\, \boldsymbol{a}$.

The isotropic elastic displacement of a screw dislocation along [0001] is given by:

$$u_z = \frac{\theta_s}{2\pi}\boldsymbol{b_s} \qquad \text{(S3)}$$

, where u_z is the atomic displacement parallel to the dislocation line, and $\boldsymbol{b_s}$ is the Burgers vector of a screw dislocation with $\boldsymbol{b_s} = \boldsymbol{c}$, and in this case of a dissociated screw partial $\boldsymbol{b_s} = ½\, \boldsymbol{c}$. And for the partial dislocations, the angle, θ_s, is defined within the range [0,2π], and the zero angle is defined for both partials along the direction opposite to the extra half-plane of the associated edge partial dislocations.

Supplementary Note 2. Image dislocations of a screw dislocation in a thin TEM foil

In the case of a screw dislocation lying parallel to a thin TEM foil, surface relaxation effects can be modelled by introducing image dislocations. Two situations are considered: the screw dislocation lying 5nm or 2.5nm below the surface of a 10nm thick foil, as shown in the Supplementary Fig. 1(a) and 1(b), respectively. A foil with two free surfaces requires an infinite series of image dislocations. Sufficient convergence was found using a series of 8 image dislocations. The largest displacements within the real sample due to these image dislocations are found to be 0.08Å and 0.25Å for the actual dislocation depths of 5nm and 2.5nm respectively. Compared to the screw displacements ½**c** of up to 2.583Å, these displacements are sufficiently small not to affect the conclusions of this work.

Supplementary Note 3. Depth of field in the corrected and uncorrected microscopes

Compared to the simulated focal series STEM ADF images of the screw dislocation using an aberration corrected microscope with a 30mrad probe convergence semi-angle (7.28nm depth of field), the simulated images using an uncorrected microscope with a 15mrad convergence semi-angle (29.1 nm depth of field) in Supplementary Fig. 2 are not able to show depth sensitive structure information. This further confirms the need for optical sectioning to observe the shear of the screw and to measure the screw Burgers vector.

The intensity of the electron probe propagating through the vacuum in 3D is calculated, and plotted along the optical axis (z-direction) at the centre of the STEM probe (see Supplementary Fig. 8). A 30mrad convergence angle (α) and 100keV beam energy (λ=0.0037nm) is used. No aberrations are included in the probe intensity simulation. An analytical formula describing the full-width at half-maximum (FWHM) of the probe along the beam direction is given by Nellist *et al* [3] as follows:

$$\delta_{\mathrm{STEM}} = \frac{1.77\lambda}{\alpha^2} \qquad \text{(S4)}$$

From Eq. S4 it can be seen that both a larger probe convergence and a shorter wavelength (higher accelerating voltage) would provide smaller depth of field and higher vertical resolution. Comparison of Supplementary Fig. 2 with Fig. 1 of the main manuscript demonstrates the improved sensitivity with reduced depth of field. This arises through both the shorter vertical length of the object that would contribute to the image at a particular focus depth, and through improved lateral resolution allowing a more precise measurement of screw displacement.

Supplementary Note 4. Focal series ADF images of [*a* + *c*] dissociated dislocations

STEM ADF optical sectioning has been applied to the [***a*** + ***c***] dissociated dislocations aligned along [0001] and viewed along $[2\bar{1}\bar{1}0]$ at a focal step of 1nm. Due to the presence of screw dislocations, the shearing of the horizontal (0002) planes along the dislocation line direction can be observed in the ADF images of the focal series. Therefore at each defocus, the shearing of (0002) planes as a function of the distance from the dislocation line can be quantified by measuring the relative shift of the intensity line profiles along the [0001] direction using a cross-correlation method. The focal series images were registered with rigid image registration prior to the cross correlation displacement measurement. The reference intensity profile was taken from the extreme right region in each ADF image which is about 7nm away from the right dislocation partial. This method is robust to local scanning distortions which are typically seen in STEM images because the cross-correlation method measures displacements from a number of (0002) planes rather than from each individual (0002) plane. In the Supplementary Fig. 4, ADF images as well as the measured displacements with the probe focused above, close to and below the dislocation plane are shown. The shearing direction of the (0002) planes is reversed from above the dislocation in the Supplementary Fig. 3a,b), to below the dislocation in the Supplementary Fig. 3e,f). The most rapid shearing occurs when the electron probe is focused close to the dislocation line in the Supplementary Fig. 3c,d), and the amount of shearing is ¼ ***c*** across each partial dislocation, suggesting that the screw component is dissociated into two partials, each with a Burgers vector of ½ ***c***.

To enhance the signal to noise and reduce distortions caused by scanning and drifting in the Figure 3 of the main text, a series of fast scanning STEM image frames were acquired from the same sample area under the same imaging condition. No visible beam damage was

noticed after 64 image frames. These 64 frames were then registered using an intensity based cross-correlation image registration method, with pure rigid shift applied to images.[4] The application of any image rotation, scaling, shearing, or non-rigid registration process was deliberately avoided due to the complicated strain conditions around the screw dislocations .The reference image for cross-correlation has been chosen as the average of the already registered images. And to increase registration accuracy the registration process has been done iteratively by updating the reference image with the new average image after each frame is registered, until the total cross-correlation value of all 64 images reaches a plateau. After the rigid registration process, 64 image frames were averaged to obtain Figure 3 in the main text.

Supplementary Note 5. Determining the dissociation distance between the two partials

Structure models with different dissociation distances were built, which contain different numbers of 4/8 rings in the fault, as shown in the Supplementary Fig. 5. The dissociation distance of the four structures are 1.10nm, 1.65nm, 2.20nm, and 2.75nm in the Supplementary Fig. 5(a-d), respectively.

The screw displacement is a function of the distance from the dislocation core. In the dissociated screw partials, the total screw displacement is given by the superposition of the screw displacements of each partial, and the total screw displacement therefore changes with changing dissociation distance, especially in regions close to the fault region. Therefore quantifying the amount of screw displacement in STEM ADF experimental and simulated images provides insight into measuring the dissociation distance. Multislice frozen phonon image simulations of ADF focal series have been performed on all four structure models of different dissociation distances shown in the Supplementary Fig. 5. These images are compared directly with the experimental images to determine the dissociation distance in the experimental image.

Instead of measuring the screw displacements directly, we have adopted the following more sensitive method by making use of the structure feature of pairs of closely-spaced column peaks in the fault region, as shown in Figure 5 of the main text. This is explained in further details here. The perfect structure of an $\boldsymbol{a}$-plane consists of successive rows of atoms along $[01\bar{1}0]$ displaced by $\frac{\sqrt{3}}{6}\boldsymbol{a}$ along $[01\bar{1}0]$ and $\boldsymbol{c}/2$ along [0001]. Close to the core, the rows of atoms above and below the core are visible, as explained in the main text. These rows above and below the screw are displaced relative to each other by the shear across the screw. The angle between $[01\bar{1}0]$ and a line joining an atom above the screw to an atom just below the

screw but belonging to the neighbouring line of atoms displaced by $\frac{\sqrt{3}}{6}\boldsymbol{a}$ along $[01\bar{1}0]$ depends on the shear across the screw, and provides a sensitive method for showing the rate at which the displacement varies across the screw.

The angles were measured using the Radon transform which is described here in detail. The Radon transform is an integral transform consisting of the projection of an image along a given projection direction [9]. The resulting one-dimensional intensity profile shows greater variance when the Radon projection is parallel to a crystalline orientation compared to when the Radon projection is slightly off, therefore allowing the image rotation to be measured based on the variance of the integrated intensity profile as a function of the Radon projection angle. One example of applying Radon transform to STEM ADF images for angle measurement can be found in Lozano *et al.*[10].

Fitting sigmoid functions (Eq. S5) to the angle plots shown in Figure 5c of the main text as well as the angle plots measured from the simulated images of all the other dissociation distances, the 1.10nm dissociation distance gives the largest value of slope-controlling parameter (parameter d in Eq.S5), and there's a monotonic increase in the slope-controlling parameter as a function of decreasing dissociation distance for a range of defocuses close to the dislocation core , as can be seen in Figure 5c of the main text. The fitted slope-controlling parameter therefore provides a robust representation of how fast the screw displacement changes across the dislocation.

$$y = a + \frac{b-a}{1+10^{(c-x)d}} \qquad \text{(S5)}$$

The robustness of the dissociation distance quantification using the sigmoid fitting method against specimen total thickness, depth of the dislocation relative to the top entrance surface,

probe defocus and probe source size is examined through ADF focal series image simulations. The simulated structure models include specimen thickness of 10nm and 15nm, dislocation depth of -5nm, -7.5nm and -10nm below the specimen top entrance surface, and source size of 0.6nm-0.9nm. The simulated images of the dissociated ½ $[a + c]$ +½ $[a + c]$ model with a dissociation distance of 1.65nm, a total specimen thickness of 15nm, the depth of the dislocation of -7.5nm is shown in the Supplementary Fig. 7(a-d) as an example. The measured slope-controlling parameters are shown in Figure 5c of the main text. Through simulations, we also find that when the specimen thickness increases to 20nm with the depth of the dislocation being -10nm, the image contrast in the Supplementary Fig. 7 (e-h) fails to match the experimental observations as shown in Figure 3 in the main text. This indicates that the specimen thickness of experiment is thinner than 20nm.

Supplementary References

1. Hirth, J. P. & Lothe, J. *Theory of Dislocations*. (Wiley, New York, 1982).

2. Moram, M. A., Barber, Z. H. & Humphreys, C. J. Accurate experimental determination of the Poisson's ratio of GaN using high-resolution x-ray diffraction. *J. Appl. Phys.* **102,** 023505 (2007).

3. Nellist, P. D., Cosgriff, E. C., Behan, G. & Kirkland, A. I. Imaging modes for scanning confocal electron microscopy in a double aberration-corrected transmission electron microscope. *Microsc. Microanal.* **14,** 82–88 (2008).

4. Yang, H. *et al.* Quantifying stoichiometry-induced variations in structure and energy of a $SrTiO_3$ symmetric sigma 13 {510}/<100> grain boundary. *Philos. Mag.* **93,** 1219–1229 (2013).

5. Béré, A. & Serra, A. Atomic structure of dislocation cores in GaN. *Phys. Rev. B* **65,** 205323 (2002).

6. Belabbas, I., Chen, J. & Nouet, G. A new atomistic model for the threading screw dislocation core in wurtzite GaN. *Comput. Mater. Sci.* **51,** 206–216 (2012).

7. Rhode, S. K. *et al.* Mg Doping Affects Dislocation Core Structures in GaN. *Phys. Rev. Lett.* **111,** 25502 (2013).

8. Hirsch, P. B. *et al.* The dissociation of the [a + c] dislocation in GaN. *Philos. Mag.* **93,** 3925–3938 (2013).

9. Deans, S. R. *The Radon Transform and Some of Its Applications*. (John Wiley & Sons, 1983).

10. Lozano, J. G. *et al.* Direct Observation of Depth-Dependent Atomic Displacements Associated with Dislocations in Gallium Nitride. *Phys. Rev. Lett.* **113,** 135503 (2014).
